# Access to free or low-cost tuberculosis treatment for migrants and refugees along the Thailand-Myanmar border in Tak province, Thailand

**DOI:** 10.1186/s12939-016-0391-z

**Published:** 2016-07-07

**Authors:** Naomi Tschirhart, Francois Nosten, Angel M. Foster

**Affiliations:** Faculty of Health Sciences, University of Ottawa, 1 Stewart Street, Ottawa, K1N 6N5 ON Canada; Shoklo Malaria Research Unit, Mahidol-Oxford Tropical Medicine Research Unit, Faculty of Tropical Medicine, Mahidol University, PO Box 46, Mae Sot, Tak 63110 Thailand; Centre for Tropical Medicine and Global Health, Nuffield Department of Clinical Medicine, University of Oxford, Churchill Hospital, Oxford, UK

**Keywords:** Healthcare access, TB treatment, Migrant, Refugee, Thailand

## Abstract

**Background:**

In Tak province, Thailand migrants and refugees from Myanmar navigate a pluralistic healthcare system to seek Tuberculosis (TB) care from a variety of government and non-governmental providers. This multi-methods qualitative study examined access to TB, TB/HIV and multidrug-resistant tuberculosis (MDR-TB) treatment with an emphasis on barriers to care and enabling factors.

**Methods:**

In the summer and fall of 2014, we conducted 12 key informant interviews with public health officials and TB treatment providers. We also conducted 11 focus group discussions with migrants and refugees who were receiving TB, TB/HIV and MDR-TB treatment in Tak province as well as non-TB patients. We analyzed these data through thematic analysis using both predetermined and emergent codes. As a second step in the qualitative analysis, we explored the barriers and enabling factors separately for migrants and refugees.

**Results:**

We found that refugees face fewer barriers to accessing TB treatment than migrants. For both migrants and refugees, legal status plays an important intermediary role in influencing the population’s ability to access care and eligibility for treatment. Our results suggest that there is a large geographical catchment area for migrants who seek TB treatment in Tak province that extends beyond provincial boundaries. Migrant participants described their ability to seek care as linked to the financial and non-financial resources required to travel and undergo treatment. Patients identified language of health services, availability of free or low cost services, and psychosocial support as important health system characteristics that affect accessibility.

**Conclusion:**

Access to TB treatment for migrants and refugees occurs at the interface of health system accessibility, population ability and legal status. In Tak province, migrant patients draw upon their social networks and financial resources to navigate a pathway to treatment. We revised a conceptual framework for access to healthcare to incorporate legal status and the cyclical pathways through which migrants access TB treatment in this region. We recommend that organizations continue to collaborate to provide supportive services that help migrants to access and continue TB treatment.

## Background

Tak province in northwestern Thailand shares an international border with Kayin (Karen) State, Myanmar. The Moei river, a narrow body of water, demarcates the border between these two countries. While the official border crossing between Mae Sot, Thailand and Myawaddy, Myanmar has a bridge, many cross the river unofficially at other locations using small boats. The border area of Tak province and Kayin state are both mountainous regions characterized by rolling hills, dusty roads, heavily forested areas and rice fields. The population of Tak province includes approximately half a million Thai citizens, 90,000 refugees, 300,000 registered migrants and an unknown number of unregistered migrants [1]. In addition to the migrant population that is living and working in the province there is significant cross-border travel among individuals who live in Myanmar but come to Thailand temporarily to access essential services, including healthcare [2]. In Tak province, five district government hospitals as well as Première Urgence - Aide Médicale Internationale (PU-AMI), the Shoklo Malaria Research Unit (SMRU), and the International Organization for Migration (IOM) deliver tuberculosis (TB) treatment to refugees and migrants from nearby Myanmar. IOM also treats TB patients on the Myanmar side of the border along with the Myanmar National TB Programme.

Previous research found a higher incidence of TB among refugees and migrants than Thai citizens living in Tak province [2]. Recent figures on the number of migrants and refugees with TB in Tak province are unavailable as the national Thai public health surveillance system does not collect data from non-governmental treatment providers. Yet, information collected from Thai government hospitals shows that for the period of 2006–2011 most of the TB cases in the province were in the Mae Sot border district and of the TB patients in the Mae Sot area more than half were non-Thais [1]. National level estimates show that Myanmar is much more heavily burdened by TB, TB/HIV and multidrug-resistant tuberculosis (MDR-TB) than Thailand [3]. In 2013, TB prevalence was higher in Myanmar (473/100,000) than Thailand (149/100,000) [3]. Estimated new cases of TB were similarly higher in Myanmar (373/100,000) than Thailand (119/100,000) [3]. In Myanmar more than a quarter (27 %) of the TB retreatment cases were multidrug resistant compared with less than one fifth (19 %) in Thailand [3]. Almost a third of the TB patients in Myanmar are co-infected with HIV compared to 15 % in Thailand [3]. Rates for TB mortality excluding TB/HIV are also four times higher in Myanmar (49/100,000) than in Thailand (12/100,000) [3].

This research project aims to examine the processes through which migrants and refugees access treatment for TB, TB/HIV and MDR-TB in Tak province as well as identify associated barriers and enabling factors. Here access is defined as “an opportunity to have health care needs fulfilled” ([4], p.4). We approach the concept of access from the perspective that it is a dynamic interplay between population needs and the health system. Previous research on access to antiretroviral therapy for migrants in Thailand identified challenges largely related to “migrants’ marginalized status in the host country”, specifically: difficulties getting health insurance and work permits, arrest by the police, treatment cost, discrimination, language, challenges leaving work to go to the clinic, social support and taking medicine at work ([5], p.1005). Telleman Saether et al. [5] identified that migrant research participants from Mae Sot had comparatively more challenges accessing healthcare than participants from Chiang Mai and Bangkok. In this article we build on previous literature by describing access to TB treatment for migrant and refugee populations and identifying the complexities associated with overlapping barriers to care [1, 2].

## Methods

### Conceptual framework

**Fig. 1:**
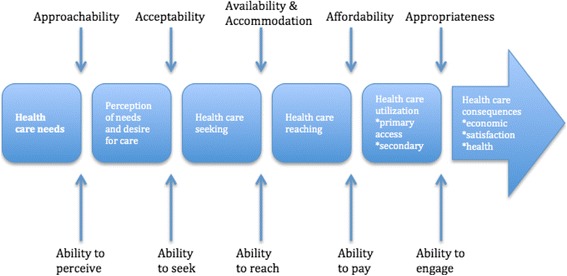
A conceptual framework of access to healthcare as developed by Levesque et al. [4]

In the field of population health conceptual frameworks are developed to help explain complex processes. A conceptual framework identifies a group of variables and describes the relationship between variables that contribute to a phenomena [6]. To examine the concept of access, we sought a theoretical framework. Access to health care models have been developed to describe the steps individuals go through to receive care as well as the supply and demand characteristics. We selected a conceptual framework developed by Levesque et al. 2013, which draws on the work of many theorists including Andersen [7] and Pechanski & Thomas [8]. The strength of Levesque et al.’s 2013 model in the context of our project on access to healthcare for migrants is that it looks at both the healthcare system and the population. Levesque and colleagues [4] conceptualize that access is related to both health care system accessibility and the ability of the patient population to interface with the system in order to gain care. Levesque et al. [4] utilize dimensions of health care system accessibility and population ability to further delineate factors that contribute to healthcare access. The five dimensions of accessibility are: 1) Approachability; 2) Acceptability; 3) Availability and accommodation; 4) Affordability; and 5) Appropriateness [4]. Ability is divided into five parallel dimensions:1) Ability to perceive; 2) Ability to seek; 3) Ability to reach; 4) Ability to pay; and 5) Ability to engage [4]. Levesque et al.’s original model is presented in Fig. 1.

### Inclusion and exclusion criteria

For this research project migrant is defined as an individual who has resided in a foreign country for more than 1 month or who has crossed a national border to access essential services, irrespective of the causes, voluntary or involuntary and the means, regular or irregular used to migrate. Individuals who have received refugee status are not considered migrants. This description is adapted and modified from a definition used by the International Organization for Migration. Documented migrant is used to describe someone who has sufficient paperwork to be able to travel legally. Undocumented migrant refers to an individual that does not have the necessary documentation. We sought migrant and refugee patients and non-patients to participate in this study. Our inclusion criteria for migrants were that they were currently living along the border, either as a migrant in Thailand or living in Myanmar and crossing into Thailand and for refugees was that they resided in a refugee camp in Tak province. Our general inclusion criteria for both groups were that they were: 20 years of age or older; sufficiently fluent in either spoken English, Burmese or Karen to participate; and willing to provide consent to participate in the study. We further disaggregated groups by gender and health status, with the respective additional inclusion criteria of: having a confirmed case of TB, MDR-TB or TB/HIV; or not having a confirmed or suspected case of TB.

We approached key informants to participate in this project who were either staff in organizations providing TB treatment or public health professionals. To be included in the study potential participants needed to be working in a care provision or policy capacity with an organization that provides TB/HIV and TB treatment to refugees and migrants in Tak province or working with the Tak provincial public health department or another organization that contributes to infectious disease surveillance. Age (20 years or older), sufficient fluency in either spoken, Thai, English, Burmese or Karen and willingness to provide consent were the additional general inclusion criteria for key informants. Participants who did not meet the inclusion criteria were excluded from the study.

### Data collection

The objective of our research project was to identify the processes through which migrants and refugees access TB treatment in Tak province and enter into the surveillance network. Qualitative methods provided us with the opportunity to explore migrants’ and refugees’ interpretation of healthcare access as well as their self-identified opportunities for action in situations where they are marginalized [9]. Collecting information from patients and health care providers allowed us to gain multiple perspectives on perceived barriers and enabling factors for TB treatment.

In the summer and fall of 2014, we collected data through key informant interviews and focus group discussions (FGD). Prior to data collection all participants consented to participate. We collected information from 12 key informants who were providing medical or supportive care to TB patients or who were working in public health in Tak province. We approached health service providers and public health surveillance specialists by email and invited them to participate in the project. Our discussion guide examined TB surveillance and treatment for migrant and refugee populations as well as organizational responsiveness to patient challenges. NT conducted the interviews in English and in Thai with the help of an interpreter.

We also held 11 FDGs with TB, TB/HIV and MDR-TB patients and four FGDs with non-patients. Table 1 provides information on the composition of the FGDs. We recruited patients from TB clinics run by SMRU, a Thai government hospital, and PU-AMI, an organization that provides TB treatment in the refugee camp. SMRU is a research unit affiliated with the Faculty of Tropical Medicine, Mahidol University, Bangkok that provides treatment services to refugee and migrant populations in Tak province. SMRU’s TB program is funded by the United Kingdom Department for International Development. Clinicians and clinic staff from all three organizations told eligible patients about the study and informed interested individuals of the time and location of the FGD. We held the FGDs in an area that was separate from where clinical services were being delivered at the Mae Sot Hospital, the refugee camp TB village and the SMRU TB village. We conducted the FGDs with non-TB patients in the refugee camp and at two community health posts in greater Mae Sot. Staff from PU-AMI and World Vision Thailand assisted in recruiting non-patient participants by informing individuals of the study as well as the time and location of the discussion. Most of the non-patients that participated in our research were also community health volunteers.

**Table 1:** Composition of focus group discussions

FGD	Location	Type	Number of participants	Description
1	Mae La TB village	Men with TB	6	Refugees and Migrants
2	Mae La TB village	A man and woman with active TB	2	Refugees
3	Mae La TB village	Women with TB	5	Refugees and migrants
4	Mae La TB village	Men who do not have TB	7	Refugees
5	Mae La TB village	Women who do not have TB	8	Refugees
6	SMRU TB village	Women with TB	5	Migrants
7	SMRU TB village	Men with TB	7	Migrants
8	SMRU TB village	Women with TB/HIV	7	Migrants
9	SMRU TB village	Men with TB/HIV	8	Migrants
10	SMRU TB village	Women with MDR-TB	6	Refugees and migrants
11	SMRU TB village	Men with MDR-TB	7	Refugee and migrants
12	Mae Sot Hospital	Women with TB	3	Migrants
13	Mae Sot Hospital	Women and Men with TB	5	Migrants
14	Community health post	Men who do not have TB	8	Migrants
15	Community health post	Women who do not have TB	8	Migrants

Our focus group discussion guide consisted of four domains of inquiry: process to access treatment, barriers, enabling resources and treatment adherence. We also utilized probes to elicit more contextual information from participants on issues related to healthcare access such as gender, language and legal status. Two interpreters assisted with the FGDs by providing simultaneous translation from Karen and Burmese languages into English.

### Data analysis

Following data collection, NT transcribed the audio files from the interviews that were done in English and two research assistants translated and transcribed the FGDs and interviews that were conducted in Karen, Burmese and Thai languages. After transcription, we analyzed the data using thematic analysis. We uploaded transcripts into the NVivo software program and subsequently coded the transcripts using both a priori and emergent codes. We reorganized the codes into themes or “implicit and explicit ideas” ([10], p.13). Data saturation for this project was reached when we ceased to identify new themes that are pertinent to the research question. We used data triangulation, the use of multiple methods to seek the same information from different perspectives, to help attain data saturation [11]. To further triangulate the data we returned to the field in June 2015 to present the preliminary results to stakeholders and to seek their feedback.

As a second step in the qualitative analysis, we identified barriers and enabling factors by population and separated the information for migrants and refugees. We then mapped the barriers and enabling factors for each population onto Levesque et al.’s [4] conceptual framework to examine the fit between our data and the framework. We identified several themes that did not fit the framework and decided to revise the model.

In reporting the results we first identify the overarching thematic domains and then we further discuss the findings by population, first reporting the findings for refugees followed by those for migrants. To help provide insight into the legal, demand and supply factors that influence migrant’s access we report according to legal status, population ability and health care system characteristics. We use personal quotes to allow the participants to share their interpretation based on their own experience while providing insight on the contextual and structural factors that shape it [9]. In order to mask identifiable information, we use pseudonyms for focus group discussion participants and identify interview participants as “key informants.”

### Ethical considerations

We obtained ethics approval from the University of Ottawa (#H02-14-08), the University of Oxford (538-14) and the Tak Provincial Public Health Office (TK 1/2557).

## Results

### Barriers and enablers for TB treatment access

**Fig. 2:**
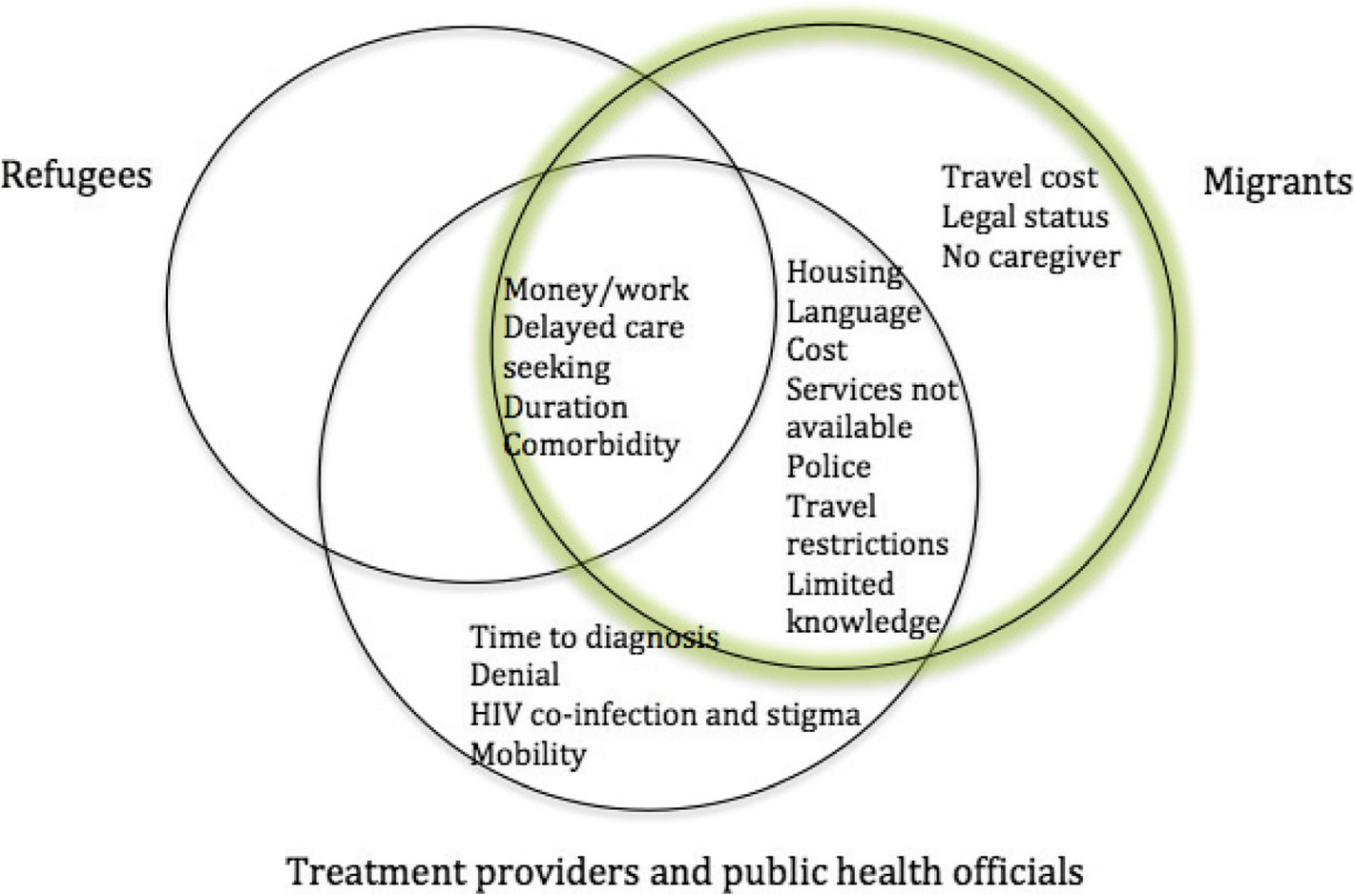
Barriers to Tuberculosis Treatment as perceived by refugees, migrants, treatment providers and public health officials

In examining barriers and enablers for TB treatment access we identified seven overarching thematic domains namely: financial, TB health services, patient health status, transport, patient beliefs and behavior, legal status and psychosocial support. A list of the barriers and enablers along with the information source is provided in Table 2. We found significant differences in access to TB treatment for refugees and migrants. Refugee participants reported fewer barriers to receiving TB treatment as compared to migrants. The barriers to TB treatment as perceived by refugees, migrants and treatment providers and public health officials, are illustrated in Fig. 2. The overlapping sections show barriers that were identified by multiple groups. For example, all groups identified these barriers: money/work, delayed care seeking, duration of treatment and comorbidity. Our key informants who provided TB treatment, support services or worked in a public health capacity identified several barriers which were not reported by patients namely: time to diagnosis, denial, patient mobility, and HIV co-infection. There was also considerable overlap in the barriers perceived by migrants and key informants, specifically: housing, language, cost, services not available, police, travel restrictions and limited knowledge.

**Table 2:** Barriers and enabling factors for migrants and refugees seeking TB treatment

Thematic Domains	Barrier	Source	Enabler	Source
Financial	Family work responsibilities	Migrant/Refugee/KI	Time off work	Migrant
	Money problems	Migrant/refugee/KI	Borrowing money	Migrant
			Community fund	Migrant
			Money	Migrant/KI
	Housing	Migrant/KI	Housing and food	Migrant/refugee/KI
TB Health services	Language	Migrant/KI	Language understood	Migrant/Refugee
			Interpreter	Migrant
	Treatment cost	Migrant/KI	Free or low cost services	Migrant/Refugee
	Services not avail	Migrant/KI	Available treatment	Migrant/Refugee
			Quality services	Migrant/Refugee
			Referral communication	Migrant/KI
			Health care workers	Migrant/Refugee
	Time to diagnosis	KI		
	Duration	Migrant/Refugee/KI		
Health status	Commorbidity (alcohol/diabetes)	Migrant/Refugee/KI		
	HIV co-infection and stigma	KI		
Transport	Travel restrictions	Migrant/KI	Transport provided by health service provider	Migrant/KI
	Police/documents	Migrant/KI	Employer transport	Migrant
	Travel cost	Migrant	Live closeby	Migrant
Patient beliefs and behaviours	Delayed care seeking	Migrant/Refugee/KI		
	Limited knowledge of TB and health system	Migrant/KI		
	Denial	KI		
	Mobility	KI		
Legal status	Undocumented	Migrant	Documents	Migrant
			Health insurance card	Migrant
Psycho social support	No caregiver	Migrant	Psychosocial activities	Migrant/KI
			Family or Community Support	Refugee/MiMigrant/KI

### Access to TB treatment for refugees

Refugees reported limited barriers to care as international organizations provide comprehensive health care free of charge in the refugee camp. PU-AMI provides TB services to the general camp population and IOM screens and treats individuals who have been accepted into the resettlement program. TB and TB/HIV treatment is provided at a residential TB village and MDR-TB cases are referred outside the camp to SMRU. PU-AMI provides transportation to SMRU for all referred patients.

Treatment at the PU-AMI TB village is provided in Burmese and Karen languages. Participants identified that provision of treatment in languages that they speak was an enabler for accessing treatment. Medics, a form of community health worker, often speak Karen and provide TB treatment in the TB village under the direction of a Myanmar doctor.

*We don't have problem here because all the staffs can speak Karen and the majority of patients use Karen language. (Bway Paw, female TB patient, refugee)*

Patients identified psychological support as an enabler to accessing and continuing treatment. In the TB village, staff hold psychosocial events to promote mental well-being among patients. Patients also described benefiting from family support.

Refugees have supportive resources including housing and food that help them to access treatment. While several refugee participants indicated concerns about the financial implications of missed work opportunities associated with TB treatment, most emphasized the buffer provided by food rations and secure housing inside the camp.

*For us as refugees, we don't have any special work and we also get free food every month. (Wiya Htoo, male TB patient, refugee)*

Key informants and refugee participants reported a few minor barriers to accessing treatment. Several refugees reported waiting until they were very ill before going to a healthcare provider for treatment. Participants also explained that co-morbidity, length of treatment for MDR-TB, stigma and denial are minor barriers to treatment for the refugee population. Comorbidity such as alcohol use may make it more difficult or complex to access healthcare. The TB camp in the refugee camp is a dry zone and drinking is prohibited. MDR-TB treatment takes 2 years and is not available in the refugee camp. Length of MDR-TB treatment may be a barrier for some patients due to the long time they would be separated from their family.

Stigma for TB is declining in the refugee camps as knowledge about the disease increases. As one KI explained that stigma for HIV is considerably higher.

*TB is infectious but it can be treated. HIV is infectious plus it can be treated but it’s a very long, very long treatment. There’s a big stigma among HIV patients inside the community. A thousand times more than TB I think. (Key informant)*

While PU-AMI provides TB treatment to the general camp population, refugees who are relocating to a third country are screened for TB by IOM. Some of the refugees who test positive for TB are in denial as they are asymptomatic. Denial may lengthen the time it takes patients to accept treatment.

### Migrant’s legal status and access to TB treatment

We found that legal status has important implications for where patients are eligible to receive free or low-cost care. Participants explained that general healthcare entitlements differed between refugees, undocumented migrants and documented migrants who had registered in the Thai migrant health insurance scheme. For example, migrants with health insurance can access TB treatment at the Thai government hospitals. In contrast, migrants without this insurance have to pay directly for treatment at the government hospitals or can access treatment free of charge where a third party donor funds their treatment.

*It depends on whether you hold any legal document or not. If you have no legal document and no health insurance then you'll have to pay but those who have it don't need to pay. (Zaw Myine, female non patient, migrant)*

A TB village run by SMRU provides free TB, TB/HIV and MDR-TB treatment to migrants regardless of legal status. SMRU also treats refugees who have MDR-TB.

Participants identified legal status as an overarching barrier for migrants from Myanmar who need to travel to access TB treatment in Thailand. Migrants without proper documents are in Thailand illegally and were concerned about their security while travelling to get treatment. Undocumented migrants are more susceptible to police extortion. On the other hand, having the correct documentation permits migrants to travel freely without concern for their personal safety. Documentation ranges from having a day pass to travel in Thailand to having a passport and work permit.

*We have a lot of problems. We have to be afraid of the police. If they catch us then we have to pay them, sometimes more than a thousand baht [USD28] and if you can't pay then they will put you in prison and then deport you. (Khin Tun, male TB patient, migrant)*

Thai police have official and unofficial checkpoints set up near the border. Migrants who lack proper travel documentation are forced to pay police money to let them through the checkpoint. Participants reported being afraid of the police. Paying police was identified by patients, non-patients and key informants as one of the major barriers to accessing TB treatment. The amount of money that police might request ranged from 100 baht (USD 3) to several thousand baht. To put this amount in context, focus group participants reported that the average daily wage of migrant workers is approximately USD3.

Migrant legal status can become more precarious if they are diagnosed with TB. Migrants undergo health screening when they are applying for their work permit or renewing it. TB testing is part of the health screening and one research participant explained that her TB status barred her from getting her work permit renewed.

*As a migrant worker we have to renew our passport visa every 2 years and every year we have to renew our work permit so we have to do health screening. My visa expired in 2014 so I needed to extend it and do the screening. When they found out that I had TB, my boss fired me and I got medication from this hospital. I have to come to this hospital every month for follow up and TB medication. (Chit Myo, female TB patient, cross border migrant)*

### Migrant’s ability to seek and reach TB treatment

While we sought to identify barriers and enablers to accessing treatment in Tak province, we discovered that there is a large geographic catchment area for migrant TB patients that extends 500 km south to Bangkok and 450 km east to Yangon, Myanmar. As the catchment area stretches beyond provincial and national boundaries, many of the factors that influence access for migrants also exist outside of the province. Some FGD participants reported complex pathways to TB treatment that began outside of Tak’s provincial jurisdiction. For example, several patients with MDR-TB who came from outside of Tak province described a lengthy search to find suitable treatment which involved seeking and in some cases receiving treatment at multiple locations. Travel is especially challenging for patients coming from rural mountainous regions of Kayin state, Myanmar as they may need to hike out of their village to seek shared motor transport to the border, hire a boat to take them across the river and then secure additional shared transport to arrive at a TB treatment provider.

We found that although most migrant patients did not initially know where to seek TB treatment in Mae Sot, this does not affect their ability to seek care as they were able to seek referral services from Mae Tao Clinic (MTC). MTC, a well know clinic that provides free healthcare services to migrants, refers TB patients to SMRU for treatment. Some of the migrant participants in our study went directly to the Thai government hospital for TB treatment.

Some of the migrant patients in the focus group discussions described waiting until they were very ill to seek treatment. Patients explained that they delayed accessing treatment due to family and work responsibilities. We found that most participants would need to take time off without pay to access treatment. Loss of income was identified as a significant barrier to treatment by TB patients who are the sole income earner in their household.

*The problem is as a man you are the head of the family and you have to work in order to get some income for your family to survive. When you come and get treatment there is a problem for daily expenses and it is not easy to go back and look after your family who live very far from you in the village. (Zaw Lwin, male MDR-TB patient, cross-border migrant)*

We found that there is a significant distinction between documented migrant workers and cross border migrants who reside in Myanmar but come across the border to Thailand for work or to access healthcare services. The distinction is important as cross border migrants are less likely to be registered as migrant workers in Thailand and are subsequently less likely to be enrolled in the migrant health insurance scheme that provides registrants with access to low cost healthcare at Thai government hospitals.

Our results suggest that access to TB treatment for migrants is closely related to available resources to reach treatment centers and pay for treatment. Migrants who have sufficient financial means can access care at the Thai government hospital and pay directly for the services.

*We have to think…overall about accommodation, treatment, food, etc. we have to think and find appropriate place to go. (Eka, female TB patient, migrant)*

We found that for migrants without travel documents, travel to reach TB treatment services is complicated by financial, logistical and legal issues. Travel costs are a barrier to accessing treatment, especially for those who are travelling a long distance from inside Myanmar. Amounts paid for transportation varied by participant and one person reported paying 150,000 kyat in travel costs (USD 135). This is significant as it represents ten percent of the GDP per capita in Myanmar which was USD 1126 in 2012 [12].

*For those who live close to this clinic for example, if they live inside Mae Sot or Myawaddy it might not cost that much for them (to travel) but imagine someone who is from very deep inside Burma, in a distant area of Karen, Kayan or Shan state, it’s a long distance to come and it will cost too much. (Aye Maung, male MDR-TB patient, cross-border migrant)*

For cross border migrants like Aye Maung it is sometimes less expensive to travel to Thailand for treatment than to venture further inland in Myanmar for care. We found that there are contextual differences for cross border migrants depending on where they need to travel through to cross over into Thailand. One participant indicated that it may be necessary to seek a guide to pass through military controlled areas in Myanmar on one’s way to Thailand.

Once in Thailand lack of documentation contributes to augment travel costs and undocumented migrants mentioned paying higher transportation costs in order to avoid Thai police checkpoints. There are travel restrictions for migrants and refugees. Migrants require a passport or day pass from Myanmar to be able travel within Tak province. Travel restrictions for refugees also affect migrants who are trying to access care in the refugee camp. In 2014, restrictions for entering and leaving the camp were more heavily enforced due to the political climate. Strict travel restrictions also make it difficult for undocumented migrants to return to clinics for follow up care. Transport provided by one’s employer or a health service provider helps migrants to access treatment without having to worry about police checkpoints or travel costs. Living close to the treatment is another enabling factor.

Migrants described the strategies that they used to gather the resources necessary to seek and obtain treatment. Some used their wages and savings or received funds from their families. Participants also reported borrowing money from friends or their bosses in order to pay for treatment. We found that flexible employers, who permitted their employees to take time off to get TB treatment, also enabled migrants’ to seek and continue treatment. Beyond financial resources and employer flexibility, migrant patients indicated that the support of family and friends as well as accommodations and food helped them to access and continue TB treatment. Families and friends provided encouragement and in some cases helped the patient search for a TB treatment provider. Having a relative in the refugee camp enabled undocumented migrants to access camp health services by coming in and staying with their relative.

*I live in the village in Burma. I went to visit my friend and her husband had TB at that time but I was healthy. After I came back home I coughed a lot so I suspected that I had TB. I have an aunt in Maela refugee camp and she asked me to come and get treatment in the camp. (Thinza, female TB patient, migrant)*

Housing and food help migrants access treatment as they consequently do not need to worry about these basic needs. SMRU has set up a TB village in Tak province which provides housing and food to patients. In addition, at the time of data collection World Vision Thailand had a community care program that provided housing and food to TB patients. PU-AMI also provides housing and a limited amount of food to migrants who seek treatment in the refugee camp.

### Accessibility of the healthcare system for Migrants

Migrants indicated that where treatment services were provided in a language that they could understand or an interpreter was present, their ability to access services was enhanced. Where health services are provided only in the Thai, language was identified as a barrier.

*I don't understand the language so I don't know what to do next after I finished the 15 days medication. I couldn't communicate so I didn't ask what disease I have at that time, I just took the medication that they gave me and realized that I feel better…. When I arrived back they told me that I had TB, I don't know which part of my body has TB, I got back pain so I thought that it might be bone TB…. The problem for me is the language because I can't speak Thai. (Lwin Aung, female TB patient, cross-border migrant)*

We found that in Tak province, availability of treatment for TB varies by legal status, comorbidity (TB/HIV) and drug resistance (MDR-TB). Migrant’s options for healthcare services narrow as TB care becomes more complex. We identified seven locations where migrants without documentation could access treatment for drug resistant TB, compared with one location for TB/HIV and one location for MDR-TB. It is important to note that SMRU is the only organization that provides TB/HIV and MDR-TB treatment to undocumented migrants. Treatment for MDR-TB is provided to migrants with health insurance at the Mae Sot district hospital. However, the amount of available funding limits the number of MDR-TB treatments that are available at the hospital and SMRU. SMRU relies on donor funding to finance its TB treatment program. At the time of this research SMRU had met the quota for MDR-TB patients and was applying for new funds.

Availability of free or low cost treatment services for TB, TB/HIV and MDR-TB in Tak province is an enabler for migrants to access treatment. While TB treatment is available to documented and undocumented migrants in Tak province, participants reported lack of affordable and accessible treatment in Bangkok and within Myanmar contributed to their decision seek care in Tak province. MDR-TB treatment is very expensive and would be unaffordable for most migrants if they had to pay for it directly. One course of treatment is 200,000 Thai baht (USD 5950). It is not only the cost for treatment that is a barrier but also the fees associated with tests necessary to determine that they have TB.

*The result came out that I couldn't continue with the medication that I was taking and MDR-TB treatment was not available in Burma that's why I came to Mae Tao Clinic. (Ye Htun, male MDR-TB patient, migrant)*

Several migrant patients reported traveling to different healthcare providers in search of effective TB treatment before coming to Tak province. Patients who had received previous TB treatment that was not effective, explained that they had to travel to get treatment. Migrants perceived treatment for TB available in Tak province is of high quality. This includes care available at the Thai government hospital, NGO healthcare providers and in the refugee camp.

*We heard that this hospital provides good treatment and services so we came here. (Mya Hla, female TB patient, cross-border migrant)*

We found that health care workers and community healthcare volunteers play an integral role in providing continuity of care through referrals, psychosocial support in addition to treatment services. Health care workers arrange comprehensive care including treatment, housing and food provision for migrant patients. In some cases they also provided patients with pocket money for living expenses. Overall patients emphasized the importance of the encouragement that the healthcare workers provided.

*While taking this medication we are really tired and sometimes we want our family's love and care but they can't come and take care of us in person. The most important people while we are here are the health care providers, they care, encourage, and help us with every single thing they can. Because of them we are still alive and have hope. (Cho Htway, female MDR-TB patient, migrant)*

We found that provision of supportive care at the community level has the potential to enhance accessibility as migrants don’t need to travel and subsequently can avoid the travel associated barriers to care. Community health volunteers live in migrant communities and provide health promotion, treatment for mild conditions and suggestions on where to access treatment. In Tak province, World Vision had a TB program which was run by community health volunteers and supported migrants through the dissemination of information about TB, locating and testing potential TB patients, and providing transport to the hospital. The program was run through community health posts in migrant communities and also provided directly observed treatment, short-course (DOTS) to patients in their community so that they could continue working.

Referrals for Migrants with TB, TB/HIV and MDR-TB are another enabler for accessing treatment. These include both official referrals through direct communication between organizations and indirect referrals where patients are told where they can get treatment. Patients are referred to treatment services in Tak province from within the province, within the country and from inside Myanmar.

## Discussion

### Overlapping perspectives on barriers to care

Our results that many of the barriers to care experienced by migrants were also identified by KIs are not surprising as the interviewees were often working closely with migrant TB patients. KIs however mentioned several barriers related to health care provision and patient characteristics, which were not cited by migrants such as patient mobility. This was identified as a concern due to the potential of drug resistance from missed doses and subsequently reduced mobility was included as an eligibility criteria for care by some care providers. We perceive that this is an important dimension of TB treatment eligibility and may deserve future inquiry.

### Integrating study results into the conceptual framework

We used the conceptual framework for access to healthcare developed by Levesque et al. [4] to organize and interpret the barriers and enabling factors that help migrants to access treatment for tuberculosis. Our results suggest that migrants’ ability to engage with the healthcare system is related to legal status, their socio-economic situation and the financial and non-financial resources that are available to them. Participants in our focus group discussions described weighing the associated costs before making a decision to seek care. Legal status has a huge influence on migrants’ ability to perceive, seek, reach, pay and engage in healthcare services. For example, our research suggests that migrants’ who do not have health insurance may not consider themselves eligible to access low-cost healthcare and Thai government hospitals. Lack of appropriate legal documentation makes it difficult to seek and reach health services as well as to return to treatment centers for follow-up care.

Levesque et al.’s framework is also useful to help conceptualize health care system accessibility for TB treatment [4]. For example, we found that referrals from partner organizations increase approachability for patients who are seeking TB treatment. Organizations in Tak province provide TB care in Burmese and Karen languages and the Thai hospital provides a translator which increases acceptability of care. In Tak province TB treatment for refugees and migrants is available, affordable and appropriate. Nonetheless we found that lack of available, inexpensive and effective TB treatment in Myanmar and Bangkok contributed to migrants’ decisions to seek treatment along the Thailand-Myanmar border.

Our results suggest that for refugee and migrant populations legal status is a factor that impacts the population’s ability to engage with the health care system in efforts to gain access to TB treatment. Legal status does not fit neatly into Levesque et al.’s framework as is not a characteristic of the population nor of the health system but plays an important role in shaping program eligibility on the supply end and affects all of the five theorized population “abilities” [4]. Given the prominence of legal status we propose to amend the framework to include legal status as an intermediary factor between population level abilities and health system accessibility. This modification makes the model more relevant to migrant and refugee contexts where legal status is an important intermediary factor that helps determine who can access care and what they can access.

**Fig. 3:**
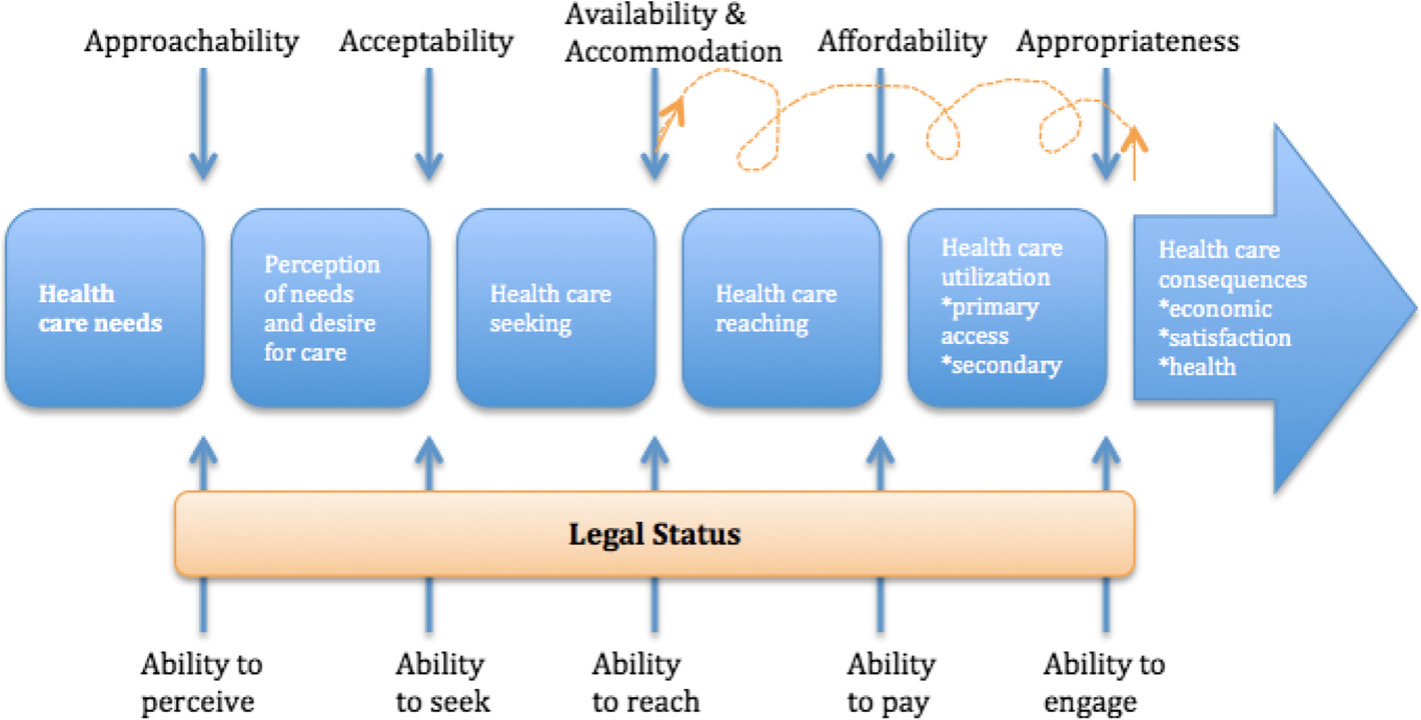
A conceptual framework for access to healthcare for migrants and refugees. Additions to the original model are shown in Orange. The arrows indicate treatment cycling as individuals find available treatment but later learn that the treatment is not appropriate and must again search for available treatment

A second challenge that we identified with the Levesque et al. framework is that as it is linear it fails to represent one of the more iterative processes that we observed [4]. Many of the migrants who participated in our project had previously received TB treatment outside of Tak province. We observed that there is a cycling in and out of treatment in search for care that is appropriate and effective. Therefore, we propose to integrate treatment cycling into the framework through a series of arrows from appropriateness to availability. Figure 3 shows the revised framework with our additions in orange.

By making legal status and treatment cycling explicit, we anticipate that this revised model can be used as an assessment tool for organizations who are delivering health services to migrant and refugee populations. Treatment providers in regions experiencing heightened migration such as Europe may find this model useful as it has the potential to help generate baseline information on treatment barriers and potential mitigating factors. Specifically, organizations can consider population ability to access care, healthcare accessibility, legal status and implications of treatment cycling in their service delivery plans. Based on the data we collected, it is evident that health service providers in Tak province, Thailand are already considering the socio-economic situation of their patients and are providing supportive care including accommodation, food and psychosocial support to help their patients complete treatment.

In further considering a population’s ability to access services, providers should consider the economic demands of treatment and the available financial and non-financial resources. We found that migrants in Tak province can access free TB treatment but still have associated economic demands such as lost wages and travel costs. Migrants’ who participated in this research identified social networks, inclusive of family members, neighbors, friends and monks, as an important resource that they utilized to enhance their ability to seek, reach and pay for care.

### Limitations

It is important to note that the migrants who participated in this study are the ones who effectively navigated the barriers to care and were successful in obtaining care in Tak province. The geo-political context along the Thailand-Myanmar border is evolving. As a result, this study’s findings should be interpreted in relation to the summer and fall 2014 data collection period. Since we completed data collection there have been changes to the government migrant health insurance scheme which we anticipate may, over time, affect access to healthcare.

## Conclusion

In Tak province, Thailand migrant and refugee’s ability to access TB treatment is complex. Access is influenced by both supply and demand characteristics within the province and beyond. Given the large geographic catchment area for patients many of the factors that influence access to treatment exist outside of the province.

Migrants who travelled from Myanmar and other locations in Thailand reported a lack of available, affordable and appropriate care in those settings. We found that migrant patients draw upon their social network, financial resources and supportive services provided by local organizations to navigate a pathway to treatment. This study is relevant for researchers and practitioners who work with migrants and refugees as it demonstrates that access to healthcare for these populations occurs at the interface of health system accessibility, population ability and legal status. Our proposed revised conceptual framework for access to healthcare, which incorporates legal status and the cyclical pathways through which migrants access care, has the potential to resonate in other contexts where legal status influences entitlement to healthcare. We recommend that treatment providers in other jurisdictions ask their patients about their pathways to treatment, identify barriers and work collaboratively to improve access to care.

## Abbreviations

FGD, Focus group discussion; IOM, International Organization for Migration; MDR-TB, multidrug-resistant tuberculosis; NGO, non-governmental organization; PU-AMI, Première Urgence - Aide Médicale Internationale; SMRU, Shoklo Malaria Research Unit; TB, tuberculosis; TB/HIV, tuberculosis and human immunodeficiency virus co-infection
